# Clinical Outcomes and Inflammatory Biomarker Changes After Intravenous Ketamine-Lidocaine Infusion in Refractory Neuropathic Pain: A Prospective Observational Pilot Study

**DOI:** 10.7759/cureus.116579

**Published:** 2026-09-20

**Authors:** Ivan Keser, Slavenka Straus, Denis Imamovic, Nermin Ismic, Anida Jamakosmanovic, Dinela Omic Salkic, Mia Sotonica, Mirko Mihalj

**Affiliations:** 1 Anesthesia and Critical Care, General Hospital “Prim. Dr. Abdulah Nakaš”, Sarajevo, BIH; 2 Clinic for Anesthesia and Reanimation, Clinical Center of the University of Sarajevo, Sarajevo, BIH; 3 Anaesthesiology, School of Medicine, University of Mostar, Mostar, BIH; 4 Anesthesiology, University Clinical Hospital of Mostar, Mostar, BIH

**Keywords:** c-reactive protein, inflammatory biomarkers, interleukin-6, intravenous infusion, ketamine, lidocaine, neuropathic pain, pain intensity

## Abstract

Background: Refractory neuropathic pain remains difficult to manage despite multimodal pharmacotherapy. Intravenous ketamine and lidocaine act through distinct analgesic mechanisms, but prospective data on their combined use and on potential inflammatory biomarker changes remain limited.

Objectives: The study aimed to evaluate changes in pain intensity and treatment tolerability following a single intravenous ketamine-lidocaine infusion in adults with refractory neuropathic pain and to explore the duration of perceived treatment effect, patient-reported functional outcomes, and changes in interleukin-6 (IL-6) and C-reactive protein (CRP).

Methods: This prospective, single-center observational pilot study included 30 adults with refractory neuropathic pain. Participants received ketamine 15 mg plus lidocaine 4 mg/kg intravenously over 30 minutes while continuing their baseline pharmacotherapy. Pain intensity was assessed using a 0-10 numerical rating scale (NRS) at baseline, immediately after infusion, and at four-week follow-up. IL-6 and CRP were measured at baseline and four weeks. Changes in NRS scores were analyzed with the Friedman test followed by Holm-adjusted Wilcoxon signed-rank tests. Biomarker changes were assessed with paired tests selected according to the distribution of within-patient differences. Adverse events and exploratory patient-reported outcomes were analyzed descriptively.

Results: Mean NRS pain intensity decreased from 7.93 ± 1.64 at baseline to 4.93 ± 2.23 immediately after infusion and 5.63 ± 2.58 at four weeks (Friedman χ²(2) = 35.50, p <0.001). Reductions from baseline were significant both immediately after infusion (adjusted p <0.001; r = 0.82) and at four weeks (adjusted p <0.001; r = 0.80), whereas the difference between immediate and four-week scores was not significant (adjusted p = 0.077). A pain reduction of at least 30% was achieved by 76.7% of patients immediately after infusion and 53.3% at four weeks; reductions of at least 50% occurred in 36.7% and 30.0%, respectively. A perceived treatment effect lasting at least three weeks or still ongoing at the four-week follow-up was reported by 56.7% of participants. IL-6 decreased from 4.73 ± 2.89 pg/mL to 4.14 ± 2.84 pg/mL (p = 0.086) and CRP from 5.51 ± 3.63 mg/L to 4.69 ± 3.71 mg/L (p = 0.120); neither biomarker correlated significantly with pain intensity. Seventy percent of patients reported no spontaneous post-infusion symptoms, no hallucinations occurred, and no adverse event required premature discontinuation or additional medical intervention.

Conclusions: A single ketamine-lidocaine infusion was followed by a reduction in self-reported pain that remained evident at four weeks in a proportion of patients, with predominantly mild and transient reported adverse effects. Because of the uncontrolled observational design, these changes cannot be attributed specifically to the pharmacological intervention, and the biomarker findings remain exploratory. Larger controlled studies with validated outcome measures and longer follow-up are needed to determine the efficacy and durability of this approach.

## Introduction

Neuropathic pain is defined as pain caused by a lesion or disease of the somatosensory nervous system and represents a clinically heterogeneous condition that can substantially impair physical functioning, psychological well-being, and quality of life [1]. Population-based studies estimate that pain with neuropathic characteristics affects approximately 7-10% of the general population [2]. Its underlying mechanisms include ectopic neuronal activity, altered ion-channel function, peripheral and central sensitization, and maladaptive changes in nociceptive processing, all of which may contribute to persistent pain despite treatment [1].

Current pharmacological management includes tricyclic antidepressants, serotonin-norepinephrine reuptake inhibitors, gabapentinoids, and selected topical therapies. However, treatment response is frequently incomplete, and adverse effects may limit dose escalation or long-term use [3]. Consequently, patients with refractory neuropathic pain may require additional therapeutic approaches when conventional multimodal treatment fails to provide adequate relief.

Intravenous ketamine and lidocaine have both been investigated as systemic analgesic therapies for refractory chronic and neuropathic pain. Ketamine is a noncompetitive N-methyl-D-aspartate receptor antagonist that may attenuate central sensitization and activity-dependent amplification of nociceptive signaling. Although intravenous ketamine has demonstrated analgesic effects in several chronic pain conditions, available evidence varies according to diagnosis, dose, and infusion protocol, and optimal patient selection remains uncertain [4]. Lidocaine primarily inhibits voltage-gated sodium channels and may reduce spontaneous ectopic discharges and neuronal hyperexcitability. Clinical studies of intravenous lidocaine in chronic neuropathic pain have reported potential analgesic benefit, although the available evidence remains heterogeneous [5].

The concurrent administration of ketamine and lidocaine has been studied less extensively than either agent alone. Their distinct pharmacological targets provide a rationale for combined administration, but evidence supporting an additive or synergistic clinical effect remains limited. A large retrospective analysis of combined ambulatory ketamine-lidocaine infusions reported pain reduction with predominantly transient adverse effects, while also demonstrating considerable variability in the magnitude and duration of individual treatment responses [6]. In addition to their analgesic actions, both agents have been associated with immunomodulatory effects. Intravenous lidocaine has been shown to influence circulating inflammatory mediators, including interleukin-6 (IL-6) and C-reactive protein (CRP), while experimental and clinical data suggest that ketamine may also modulate pro-inflammatory cytokine signaling [7,8]. The clinical relevance of these inflammatory changes in patients with chronic neuropathic pain, however, remains uncertain.

The primary objective of this prospective observational pilot study was to evaluate changes in pain intensity following a single intravenous ketamine-lidocaine infusion in adults with refractory neuropathic pain. Secondary objectives were to describe the duration of analgesic response and treatment-related adverse effects and to explore changes in patient-reported emotional and functional status and inflammatory biomarkers, including IL-6 and CRP, during four weeks of follow-up. Preliminary findings from this study were previously presented at the 20th Belgrade International Symposium on Pain (BISOP), held in Belgrade, Serbia, on May 17, 2025, and published in the conference proceedings.

## Materials and methods

Participants

This prospective, single-center observational pilot study was conducted at the General Hospital “Prim. Dr. Abdulah Nakaš” in Sarajevo, Bosnia and Herzegovina, following Ethics Committee approval in March 2025. The study was conducted from March 2025 to May 2025. The study included 30 adult patients with refractory neuropathic pain who received intravenous ketamine-lidocaine infusion as part of routine clinical management. The present cohort represented the predefined pilot phase of a larger planned observational study; enrollment was not continued beyond the pilot phase. The decision not to continue enrollment beyond the predefined pilot cohort was made for logistical and organizational reasons before the study outcome data were analyzed and was not based on the observed treatment response. Patients were followed from baseline through a four-week post-infusion assessment.

The pilot sample size was predefined as 30 participants; no formal power calculation was performed for this exploratory pilot phase.

Intravenous ketamine-lidocaine infusion was part of routine clinical management for selected patients with refractory neuropathic pain and was not administered solely for research purposes. The clinical decision to administer ketamine-lidocaine infusion was made before and independently of study enrollment. Consecutive patients for whom this treatment had been selected as part of routine clinical care were assessed for study eligibility, and those who met the predefined inclusion criteria and provided written informed consent were enrolled. The study prospectively evaluated clinical outcomes following this treatment without altering the patients’ baseline pharmacological therapy.

For the purposes of this study, refractory neuropathic pain was defined as clinically diagnosed neuropathic pain, confirmed by a Douleur Neuropathique 4 (DN4) score ≥4, with persistent inadequate symptom relief despite previously prescribed standard pharmacological treatment [9]. Because detailed treatment histories were not collected with sufficient granularity to define refractoriness by a prespecified number, dose, or duration of failed drug trials, refractoriness was operationalized using the above clinical criteria. The ketamine-lidocaine infusion was administered as an adjunct to the patients’ existing treatment regimen; previously prescribed pharmacotherapy was not discontinued as part of the study protocol.

Eligible participants were adults aged >18 years with refractory neuropathic pain confirmed using the DN4 questionnaire, with a score of ≥4 considered consistent with neuropathic pain [9], inadequate symptom relief despite standard pharmacological treatment, and written informed consent for participation.

Exclusion criteria were known hypersensitivity or contraindication to ketamine or lidocaine, pregnancy or breastfeeding, clinically significant cardiovascular disease, including unstable angina or poorly controlled hypertension, elevated intracranial or intraocular pressure, and a history of psychosis or other severe psychiatric disorders.

Clinical assessment

At baseline, participants completed a structured study-specific questionnaire developed by the investigators to characterize neuropathic pain symptoms, pain frequency and intensity, functional limitations, energy and motivation, social functioning, anxiety or nervousness, and perceived stress. Pain intensity was recorded using an 11-point numerical rating scale (NRS), ranging from 0 (no pain) to 10 (worst imaginable pain), with higher scores indicating greater pain intensity [10].

The questionnaire was developed specifically for this study and had not undergone external psychometric validation. Therefore, questionnaire items concerning emotional and functional status were treated as patient-reported exploratory outcomes rather than as validated measures of depression, anxiety, or quality of life. The complete study-specific questionnaire is provided in Appendix A.

Infusion protocol and monitoring

Before administration of the infusion, patients underwent clinical assessment and standard physiological monitoring, including electrocardiography, non-invasive blood pressure measurement, heart rate monitoring, and peripheral oxygen saturation.

A single intravenous infusion containing ketamine at a fixed dose of 15 mg and lidocaine at a dose of 4 mg/kg was administered over 30 minutes. Patients were continuously monitored during the infusion and observed clinically for one hour after its completion. Blood pressure and heart rate were recorded before and after the procedure, and any clinically relevant hemodynamic changes or acute adverse events were documented.

All participants continued their previously prescribed pharmacological treatment for neuropathic pain during the study period. Concomitant pharmacological treatment remained unchanged during the four-week follow-up, with no initiation, discontinuation, or dose adjustment of analgesic medications and no additional interventional pain procedures. The ketamine-lidocaine infusion was used as an adjunctive intervention and did not replace baseline therapy.

Outcome assessment

Pain intensity was assessed at baseline, immediately after completion of the infusion, and at the four-week follow-up visit. The immediate post-infusion NRS assessment and the structured sedation/alertness assessment were recorded as separate study measures during the immediate post-infusion period. All participants were able to provide an immediate post-infusion NRS score; however, the exact temporal relationship between the NRS assessment and subsequent changes in alertness was not recorded with sufficient granularity to determine whether reduced alertness overlapped with pain assessment in individual patients. At follow-up, participants were also asked to characterize the perceived change in pain and the duration of the therapeutic effect.

The primary outcome was the change in NRS pain intensity at baseline, immediately post-infusion, and four-week follow-up assessment. Secondary and exploratory outcomes included treatment tolerability, perceived duration of treatment effect, patient-reported functional and emotional outcomes, and changes in IL-6 and CRP.

Treatment tolerability and safety were assessed by recording patient-reported adverse effects and specific post-infusion symptoms, including nausea, hallucinations, and changes in alertness or sedation. Patient-reported changes in everyday functioning, emotional stability, perceived calmness, general well-being, and satisfaction with pain control were also recorded at follow-up.

Because the study-specific questionnaire was not a validated psychometric instrument, these measures were analyzed as individual exploratory patient-reported outcomes and were not interpreted as formal diagnostic assessments of depression, anxiety, or health-related quality of life.

Laboratory assessment

Venous blood samples were obtained at baseline before the infusion and again at the four-week follow-up visit. For IL-6 measurement, blood was collected into serum tubes, and serum was separated within one hour of collection and stored at a temperature below -20°C until analysis. IL-6 concentrations were measured using the commercially available ADVIA Centaur® IL6 assay (Siemens Healthcare Diagnostics Inc., Tarrytown, USA) by chemiluminescent immunoassay on an ADVIA Centaur® XPT analyzer (Siemens Healthcare Diagnostics Inc., Tarrytown, USA), according to the manufacturer’s instructions. CRP concentrations were measured using a Dimension® EXL 200 Integrated Chemistry System (Siemens Healthcare Diagnostics Inc., Newark, USA), according to the manufacturer’s instructions. Changes in IL-6 and CRP between baseline and four weeks were evaluated as exploratory secondary outcomes, and associations between each biomarker and pain intensity were also explored.

Ethical considerations

The study was conducted in accordance with the principles of the Declaration of Helsinki and was approved by the Ethics Committee of the General Hospital “Prim. Dr. Abdulah Nakaš,” Sarajevo, Bosnia and Herzegovina (approval No. 26-213-147/25). Approval was granted at the committee meeting held on March 5, 2025, and the official approval letter was issued on March 10, 2025. Written informed consent was obtained from all participants before enrollment. All study data were analyzed in anonymized form.

Statistical analysis

Continuous variables were summarized using mean and standard deviation (SD) and, where appropriate, median and interquartile range (IQR), while categorical variables were presented as frequencies and percentages. The distribution of continuous variables was assessed using the Shapiro-Wilk test.

Pain intensity was assessed on a 0-10 NRS at three time points: before infusion, immediately after infusion, and four weeks after infusion. Because pain scores were not normally distributed, changes across the three repeated measurements were evaluated using the Friedman test. When the overall test was significant, pairwise comparisons were performed using the Wilcoxon signed-rank test with Holm adjustment for multiple comparisons. Effect sizes for pairwise comparisons were calculated as follows: $r = \frac{|Z|}{\sqrt{n}}$.

As exploratory measures of clinical response, the proportions of patients achieving ≥30% and ≥50% reductions in NRS pain intensity from baseline were calculated immediately after infusion and at the four-week follow-up, consistent with commonly used thresholds for clinically important improvement in chronic pain [11].

Changes in inflammatory biomarkers between baseline and four-week follow-up were analyzed using paired tests according to the distribution of within-patient differences. IL-6 was analyzed using a paired-samples t-test, whereas CRP was analyzed using the Wilcoxon signed-rank test. Associations between pain intensity and inflammatory biomarkers were explored using Spearman rank correlation coefficients.

Adverse events, duration of perceived therapeutic effect, and patient-reported changes in emotional status, daily functioning, general well-being, and satisfaction with pain control were analyzed descriptively and reported as numbers and percentages. Because these outcomes were obtained using a study-specific, non-validated questionnaire, no inferential analyses were performed to establish formal changes in depression, anxiety, or health-related quality of life.

No missing-value imputation was performed. All available paired observations were included in the corresponding analyses. All statistical tests were two-sided, and a p-value <0.05 was considered statistically significant. Statistical analyses were performed using IBM SPSS Statistics version 26.0 (IBM Corp., Armonk, USA).

## Results

Baseline characteristics

A total of 30 patients with refractory neuropathic pain were included in the analysis. All 30 participants completed the four-week follow-up and were included in the paired analyses. The study population comprised 21 women (70.0%) and nine men (30.0%), with a mean age of 48.7 ± 10.1 years (range, 27-73 years). Baseline pain intensity was high, with a mean NRS score of 7.93 ± 1.64 and a median of 8 (IQR, 6-10). Tingling or paresthesia was reported by 28 patients (93.3%), while 24 (80.0%) described an exaggerated painful response to stimulation. Functional impairment was common, with 18 patients (60.0%) reporting severe limitation of daily activities. Baseline demographic and clinical characteristics are summarized in Table 1.

**Table 1:** Baseline demographic and clinical characteristics of the study population Values are presented as n (%) unless otherwise indicated. NRS, numerical rating scale; SD, standard deviation; IQR, interquartile range.

Characteristic	Value
Participants, n	30
Age, years, mean ± SD	48.7 ± 10.1
Age range, years	27-73
Female sex	21 (70.0%)
Male sex	9 (30.0%)
Baseline NRS, mean ± SD	7.93 ± 1.64
Baseline NRS, median (IQR)	8 (6-10)
Burning pain	13 (43.3%)
Stabbing pain	10 (33.3%)
Pressure-like pain	7 (23.3%)
Tingling or paresthesia	28 (93.3%)
Pain with touch or mild pressure	12 (40.0%)
Exaggerated pain response to stimulation	24 (80.0%)
Severe limitation of daily activities	18 (60.0%)
Some limitation of daily activities	11 (36.7%)
No limitation of daily activities	1 (3.3%)

Pain intensity outcomes

Pain intensity decreased following ketamine-lidocaine infusion. The mean NRS score decreased from 7.93 ± 1.64 at baseline to 4.93 ± 2.23 immediately after infusion and remained lower at 5.63 ± 2.58 at the four-week follow-up. Median NRS scores were 8 (IQR, 6-10), 4.5 (IQR, 3-6), and 5 (IQR, 4-7.5), respectively. The overall change in pain intensity across the three assessment time points was statistically significant (Friedman χ²(2) = 35.50, p <0.001) (Figure 1).

**Figure 1:**
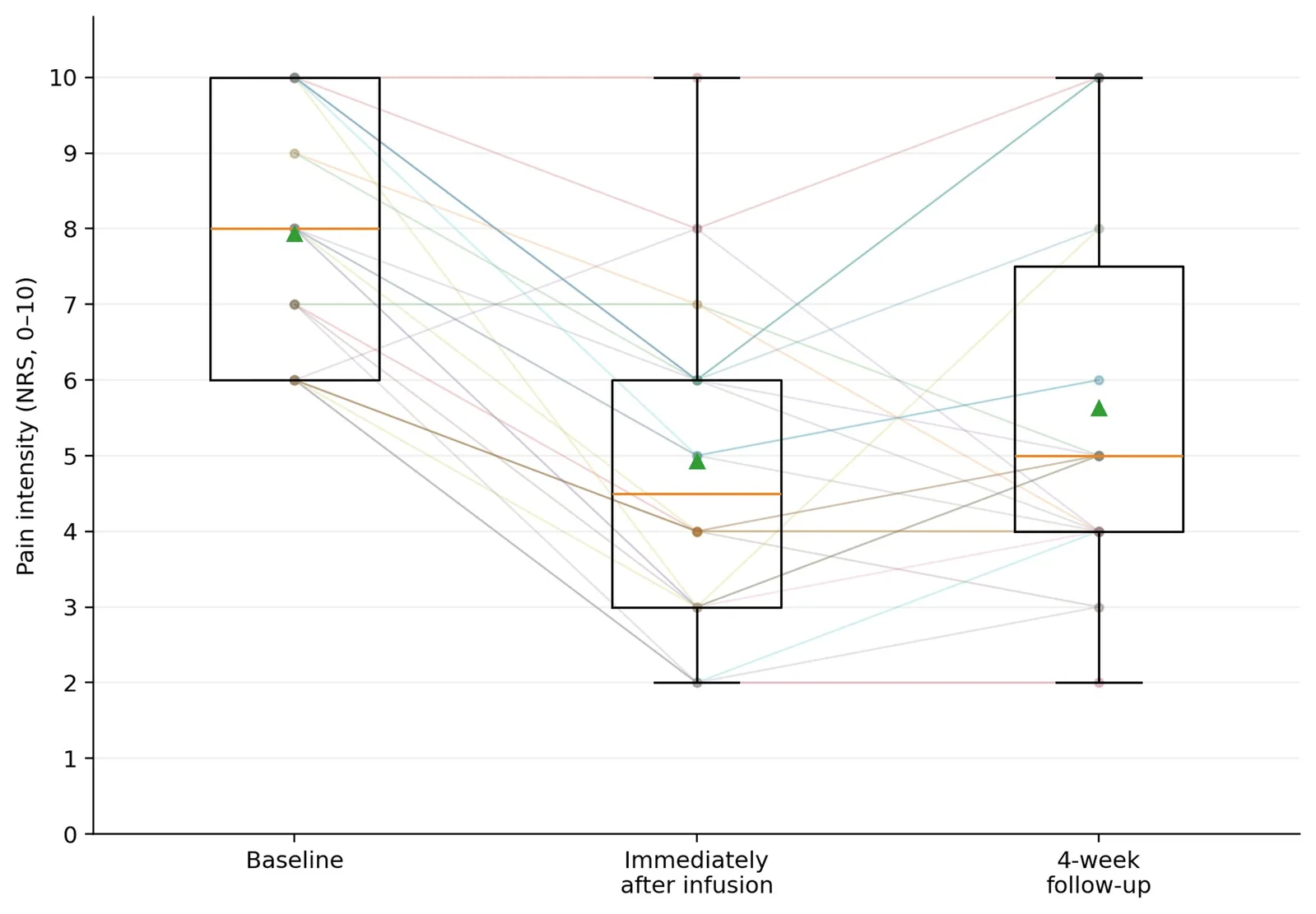
Pain intensity at baseline, immediately after ketamine-lidocaine infusion, and at four-week follow-up Individual patient trajectories and box-and-whisker plots showing pain intensity measured using the 0-10 NRS at baseline, immediately after infusion, and at four-week follow-up. Boxes represent the interquartile range, horizontal lines represent medians, and triangular markers represent means. NRS, numerical rating scale.

Post-hoc Wilcoxon signed-rank tests with Holm adjustment demonstrated significant reductions from baseline to immediately after infusion and from baseline to the four-week follow-up, both with large effect sizes. The difference between the immediate post-infusion and four-week measurements was not statistically significant. Detailed pairwise comparisons are presented in Table 2.

**Table 2:** Pairwise comparisons of pain intensity following ketamine-lidocaine infusion Pairwise comparisons were performed using Wilcoxon signed-rank tests with Holm adjustment for multiple comparisons following a significant Friedman test (χ²(2) = 35.50, p <0.001). Effect sizes were calculated as described in the 'statistical analysis' section. A p-value <0.05 was considered statistically significant.

Comparison	Z statistic	Holm-adjusted p-value	Effect size (r)
Baseline vs immediately after infusion	-4.47	<0.001	0.82
Baseline vs four-week follow-up	-4.36	<0.001	0.80
Immediately after infusion vs four-week follow-up	-1.77	0.077	0.32

As an exploratory assessment of treatment response, 23 of 30 patients (76.7%) achieved a reduction of at least 30% in pain intensity immediately after infusion, while 16 patients (53.3%) achieved a reduction of at least 30% at four weeks. A reduction of at least 50% was observed in 11 patients (36.7%) immediately after infusion and nine patients (30.0%) at four weeks. No changes in baseline analgesic therapy and no additional pain interventions were recorded during the four-week follow-up.

Duration of therapeutic effect and exploratory patient-reported outcomes

The perceived duration of treatment effect varied among participants. Nine patients (30.0%) reported that the treatment effect was still ongoing at the four-week follow-up, eight (26.7%) reported an effect lasting three to four weeks, six (20.0%) reported an effect lasting one to two weeks, and seven (23.3%) reported an effect lasting less than one week. Overall, 17 of 30 patients (56.7%) reported an effect lasting at least three weeks or still ongoing at the four-week follow-up.

At the four-week follow-up, improvement in perceived pain control was reported by 10 patients (33.3%), improvement in daily activity by 12 (40.0%), and improvement in general well-being by 16 (53.3%). Because these outcomes were obtained using a study-specific, non-validated questionnaire, they were considered exploratory and analyzed descriptively.

Inflammatory biomarkers

Mean IL-6 concentration decreased from 4.73 ± 2.89 pg/mL at baseline to 4.14 ± 2.84 pg/mL at the four-week follow-up. The distribution of within-patient differences was compatible with normality, and the change was not statistically significant on paired-samples testing (t(29) = 1.78, p = 0.086).

Mean CRP concentration decreased from 5.51 ± 3.63 mg/L at baseline to 4.69 ± 3.71 mg/L at four weeks. Because the distribution of paired differences was non-normal, the Wilcoxon signed-rank test was applied; the observed change was not statistically significant (W = 157.0, Z = -1.55, p = 0.120). Changes in inflammatory biomarkers are summarized in Table 3.

**Table 3:** Changes in inflammatory biomarkers from baseline to four-week follow-up A p-value <0.05 was considered statistically significant. IL-6, interleukin-6; CRP, C-reactive protein; SD, standard deviation.

Biomarker	Baseline, mean ± SD	Four-week follow-up, mean ± SD	Statistical test	Test statistic	p-value
IL-6, pg/mL	4.73 ± 2.89	4.14 ± 2.84	Paired-samples t-test	t(29) = 1.78	0.086
CRP, mg/L	5.51 ± 3.63	4.69 ± 3.71	Wilcoxon signed-rank test	W = 157.0; Z = -1.55	0.120

Exploratory correlation analyses did not demonstrate significant associations between pain intensity and inflammatory biomarkers. At baseline, pain intensity was not significantly correlated with IL-6 (Spearman ρ = 0.242, p = 0.197) or CRP (ρ = 0.214, p = 0.257). Similarly, no significant correlations were observed at four weeks for IL-6 (ρ = 0.013, p = 0.944) or CRP (ρ = -0.083, p = 0.663).

Safety and tolerability

During post-infusion assessment, 21 of 30 patients (70.0%) reported no spontaneous adverse symptoms. Nine patients (30.0%) reported a spontaneous post-infusion symptom: drowsiness in four patients (13.3%), dizziness in two (6.7%), nausea accompanied by headache in two (6.7%), and a transient electric-shock sensation in one patient (3.3%). No hallucinations were reported.

On the study-specific assessment of alertness, 21 patients (70.0%) reported no sedation, seven (23.3%) reported moderately reduced alertness, one (3.3%) reported mild drowsiness, and one (3.3%) selected the “severe sedation” response category. All participants, including this patient, provided an immediate post-infusion NRS score. The patient who selected the “severe sedation” response category remained under clinical observation and did not require additional medical intervention. On the separate structured nausea assessment, mild nausea was reported by one patient (3.3%), while 29 patients (96.7%) reported no nausea. Safety and tolerability findings are summarized in Table 4.

**Table 4:** Safety and tolerability following ketamine-lidocaine infusion Spontaneously reported symptoms and structured assessments of sedation, nausea, and hallucinations represent separate questionnaire items and therefore should not be summed across categories.

Assessment	Finding	n (%)
Spontaneous symptoms	None	21 (70.0%)
	Drowsiness	4 (13.3%)
	Dizziness	2 (6.7%)
	Nausea with headache	2 (6.7%)
	Electric-shock sensation	1 (3.3%)
Sedation/alertness	No sedation	21 (70.0%)
	Mild drowsiness	1 (3.3%)
	Moderately reduced alertness	7 (23.3%)
	Severe sedation response category	1 (3.3%)
Structured nausea assessment	No nausea	29 (96.7%)
	Mild nausea	1 (3.3%)
Hallucinations	None reported	30 (100%)

No clinically relevant changes in blood pressure, heart rate, oxygen saturation, or cardiac rhythm were observed during the infusion or the immediate post-infusion monitoring period. No adverse event required premature discontinuation of the infusion or additional medical intervention.

## Discussion

The principal finding of this prospective observational pilot study was a significant reduction in pain intensity following a single intravenous ketamine-lidocaine infusion in patients with refractory neuropathic pain. Mean NRS pain intensity decreased from 7.93 at baseline to 4.93 immediately after infusion and remained lower at 5.63 at the four-week follow-up. The reductions from baseline were statistically significant and were accompanied by large effect sizes. In addition, 76.7% of participants achieved at least a 30% reduction in pain immediately after infusion, and 53.3% achieved this level of response at four weeks. Although the absence of a control group prevents attribution of these changes solely to the infusion, the persistence of lower pain scores at four weeks suggests that the observed response was not limited to the immediate post-infusion period.

These findings are broadly consistent with the available evidence supporting the analgesic potential of intravenous ketamine in neuropathic pain. A systematic review and meta-analysis of randomized controlled trials found that ketamine was associated with reductions in neuropathic pain intensity, although considerable heterogeneity existed with respect to underlying diagnoses, dosing strategies, infusion duration, and follow-up periods [12]. Similar conclusions were reported in a more recent systematic review examining ketamine in patients with multifactorial neuropathic pain, which suggested potential analgesic benefit while emphasizing variability among treatment protocols and the need for better-defined patient selection and dosing strategies [13]. The present study differs from many previous investigations in that a relatively low fixed dose of ketamine was administered together with weight-based intravenous lidocaine rather than ketamine being evaluated as an isolated intervention.

The potential contribution of lidocaine should therefore also be considered. Intravenous lidocaine reduces neuronal excitability primarily through inhibition of voltage-gated sodium channels and has been investigated in a range of chronic neuropathic pain conditions. A systematic review by Lee et al. found evidence of analgesic benefit following intravenous lidocaine, although the magnitude and duration of effect varied considerably among studies [5]. Similarly, current consensus recommendations regarding intravenous ketamine emphasize substantial variation in protocols and highlight the importance of individualized patient selection, appropriate monitoring, and careful evaluation of treatment response [4]. Because ketamine and lidocaine act through different pharmacological mechanisms, their concurrent administration may provide a rational multimodal approach. However, the present observational design cannot establish whether the observed response resulted predominantly from ketamine, lidocaine, their combined administration, or other nonspecific treatment effects.

Evidence specifically evaluating combined ketamine-lidocaine infusion remains limited. Striebel et al. reported outcomes from ambulatory infusion therapy using simultaneous ketamine and lidocaine and observed reductions in pain intensity in a substantial proportion of treated patients, with predominantly transient adverse effects [6]. Their findings also demonstrated marked interindividual variability in treatment response, which is consistent with the variability observed in the present cohort. More than half of participants reported an effect lasting at least three weeks or still ongoing at the four-week follow-up, while others experienced a substantially shorter duration of benefit. This variation may reflect differences in neuropathic pain mechanisms, underlying diagnoses, disease duration, concomitant pharmacotherapy, and individual pharmacodynamic responses. Identification of clinical characteristics associated with prolonged response may therefore represent an important objective for future studies.

Exploratory patient-reported outcomes also suggested improvement beyond pain intensity alone. At four weeks, 40.0% of participants reported improvement in daily activity and 53.3% reported improvement in general well-being. Chronic pain is closely associated with functional impairment and psychological distress, and the relationship between pain and emotional symptoms is bidirectional [14]. Ketamine has also been extensively studied for rapid effects on depressive symptoms through mechanisms that extend beyond conventional monoaminergic pathways [15]. Nevertheless, the emotional and functional questions used in the present study were derived from a study-specific questionnaire that had not undergone formal psychometric validation. These findings should therefore be interpreted as exploratory patient-reported observations rather than evidence of a specific antidepressant, anxiolytic, or quality-of-life effect.

An additional objective of this study was to explore whether clinical improvement was accompanied by measurable changes in inflammatory biomarkers. Mean IL-6 and CRP concentrations were numerically lower at four weeks than at baseline, but neither change reached statistical significance. Furthermore, neither biomarker demonstrated a significant correlation with pain intensity at baseline or follow-up. These findings do not support a measurable systemic anti-inflammatory effect of the intervention in this cohort. Previous studies have reported immunomodulatory effects associated with intravenous lidocaine, including changes in circulating inflammatory mediators [7], while experimental and clinical evidence suggests that ketamine may influence pro-inflammatory cytokine signaling [8]. However, inflammatory responses are influenced by numerous clinical and biological factors, and a single follow-up measurement at four weeks may not capture transient changes occurring shortly after drug administration. Accordingly, the present findings should be interpreted as showing no significant change in IL-6 or CRP at four weeks and should not be taken as evidence against a transient inflammatory effect occurring earlier after the infusion. The small sample size also limits the ability to detect modest biomarker effects.

The infusion was generally well-tolerated under monitored conditions. Seventy percent of participants reported no spontaneous post-infusion adverse symptoms, while the most frequently reported symptoms were drowsiness, dizziness, and nausea accompanied by headache. No hallucinations were reported, and no adverse event required premature discontinuation of the infusion or additional medical intervention. These observations are consistent with the known short-term adverse effect profile of intravenous ketamine and with previous experience using combined ketamine-lidocaine infusion [4,6]. Nevertheless, the study was not powered to establish safety, and the relatively small sample size does not permit conclusions regarding uncommon or serious adverse events. The findings should therefore not be interpreted as demonstrating the safety of this regimen outside an appropriately monitored clinical environment.

Strengths and limitations

The principal strengths of this study include its prospective design, standardized infusion protocol, repeated assessment of pain intensity, complete four-week follow-up of the pilot cohort, and simultaneous evaluation of clinical response, treatment tolerability, and inflammatory biomarkers. Reporting both continuous changes in NRS scores and the proportions of patients achieving at least 30% and 50% pain reduction provides complementary information regarding the magnitude of the observed clinical response.

Several limitations should be considered. First, this was a small, single-center pilot study involving 30 participants, which limits statistical power and generalizability. Second, the absence of a placebo or active control group prevents determination of the extent to which improvements resulted specifically from ketamine-lidocaine infusion rather than placebo effects, regression to the mean, natural variation in pain intensity, or continued baseline pharmacotherapy. Third, ketamine and lidocaine were administered concurrently, precluding assessment of the independent contribution of either drug or demonstration of an additive or synergistic effect. The fixed ketamine dose of 15 mg was relatively low compared with many previously reported intravenous ketamine protocols, which may limit comparability with studies using higher weight-based doses [4,12]. Fourth, participants had refractory neuropathic pain representing a clinically heterogeneous population, and the sample size was insufficient for meaningful subgroup analyses according to pain etiology or other potential predictors of response. Detailed information regarding specific underlying neuropathic pain diagnoses, pain duration, and the classes, doses, duration, and adequacy of individual previous pharmacological trials was not systematically collected, limiting further characterization of treatment refractoriness and clinical heterogeneity. Fifth, follow-up was limited to four weeks, preventing assessment of longer-term efficacy, repeated-treatment effects, or cumulative toxicity. Sixth, functional and emotional outcomes were assessed using a study-specific, non-validated questionnaire and should therefore be regarded as exploratory. Because the exact timing of the structured alertness assessment relative to the immediate post-infusion NRS was not recorded for each participant, a potential influence of acute drug-related changes in alertness on the immediate pain rating cannot be completely excluded. Finally, inflammatory biomarkers were measured only at baseline and at four weeks; transient biological changes occurring during the hours or days following infusion may consequently have been missed.

Despite these limitations, the observed reduction in pain intensity and the persistence of lower pain scores at four weeks provide a clinically relevant signal that warrants further investigation. Future randomized controlled trials should compare combined ketamine-lidocaine therapy with placebo and, ideally, with ketamine-only and lidocaine-only treatment arms. Larger samples would also permit evaluation of predictors of treatment response, dose-response relationships, optimal infusion schedules, and the characteristics of patients most likely to experience sustained benefit. The use of validated measures of physical functioning, emotional health, and quality of life, together with more frequent biomarker sampling and longer follow-up, would provide a more comprehensive assessment of both clinical and biological treatment effects.

## Conclusions

A single ketamine-lidocaine infusion was followed by a reduction in self-reported pain that persisted at four weeks in a proportion of patients, with predominantly mild and transient reported adverse effects. The uncontrolled design prevents attributing these changes specifically to the pharmacological intervention, and the biomarker findings remain exploratory. Larger controlled studies with validated outcome measures and longer follow-up are needed to determine the efficacy and durability of this approach.

## Appendices

Appendix A: Study-specific questionnaire

This investigator-developed questionnaire was originally administered in Bosnian and was translated into English for publication. The questionnaire has not undergone formal psychometric validation. Identifying information collected on the original study form has been omitted from the published version.

Part I: Baseline Assessment

1. Date: __________

2. Have you visited a physician because of pain during the past month? Yes No I have a scheduled appointment

3. How satisfied are you with your current pain treatment? Very satisfied Satisfied No change noticed The treatment has not helped Very dissatisfied

4. How would you describe the nature of your pain? Burning Stabbing Sharp pain Pressure or squeezing sensation Other (please specify): __________

5. How often do you experience pain? Constantly Intermittently, in episodes Only in certain situations (e.g., during movement)

6. What is the intensity of your pain during the recent period? Please rate your pain on an 11-point numerical rating scale (NRS):

0 | 1 | 2 | 3 | 4 | 5 | 6 | 7 | 8 | 9 | 10

0 = No pain; 10 = Worst pain imaginable

7. How would you describe the pain sensation? I experience pain, but it does not feel like pain from a typical injury. I experience pain that is much stronger than would normally be expected from an injury. I experience pain that lasts longer than would normally be expected.

8. Do you experience tingling or pins-and-needles sensations in certain parts of your body? Yes No

9. Do you experience pain caused by touch or mild pressure, such as contact with clothing or bed linen? Yes No

10. Do you experience pain that is stronger than the normal response to a stimulus? Yes No

11. How would you describe your ability to perform daily activities because of pain? No problems performing daily activities Some difficulty, but I am able to perform basic activities Severe difficulty performing daily activities

12. During the past seven days, how would you describe your level of energy for daily activities? I have more energy than usual My energy level is approximately the same as usual I feel tired and exhausted I have almost no energy

13. During the past seven days, have you felt less motivated to do things you usually enjoy? No, I still have a high level of motivation I occasionally feel less motivated I often lack motivation to do things I usually enjoy I have no motivation to do anything

14. How satisfied have you been with your social life during the past seven days? I feel very connected with other people I have had some contact with friends and family I feel somewhat lonely I have had almost no social interaction

15. How often have you experienced anxiety or nervousness during the past seven days? Never Occasionally Often Almost every day

16. How often have you felt under significant stress during the past seven days? Never Occasionally Often Almost every day

Part II: Follow-up After Ketamine-Lidocaine Infusion

The pain-intensity NRS item (Item 2) was administered immediately after completion of the infusion and was repeated at the four-week follow-up. Questions concerning the perceived duration of treatment effect and longer-term patient-reported outcomes were completed at the four-week follow-up.

1. Have you noticed any change in pain intensity following the infusion? Substantial reduction in pain Mild reduction in pain No change Increased pain

2. What is the intensity of your pain at the time of this assessment? Please rate your pain on an 11-point numerical rating scale (NRS):

0 | 1 | 2 | 3 | 4 | 5 | 6 | 7 | 8 | 9 | 10

0 = No pain; 10 = Worst pain imaginable

3. How long did the perceived effect of the infusion last? Less than one week One to two weeks Three to four weeks More than four weeks

For analysis, the response category “More than four weeks” was interpreted as indicating that the perceived treatment effect was still ongoing at the four-week follow-up, because no assessment beyond four weeks was performed.

4. Did you experience any adverse effects following the infusion? Yes (please specify): __________ No

5. How would you rate your overall quality of life following the infusion? Substantial improvement Mild improvement No change Worsening

6. Did you experience hallucinations following the infusion, such as seeing or hearing something that was not actually present? Yes No Unsure

7. How would you describe your level of sedation or drowsiness following the infusion? No sedation Mild drowsiness Moderately reduced alertness Severe sedation (difficulty remaining awake)

8. Did you experience nausea following the infusion? Severe nausea Mild nausea No nausea

9. How would you describe your emotional stability during the past several days? I feel emotionally stable I occasionally feel emotionally unsettled I feel emotionally unstable I feel completely overwhelmed

10. Did you feel calm and relaxed following the infusion? Yes, I feel much calmer Slightly calmer No change Worsening; I feel more tense

11. How satisfied are you with your progress in pain control following the infusion? Substantial improvement Mild improvement No change Worsening

12. How would you describe changes in your daily activities following the infusion? Substantial improvement Mild improvement No change Worsening

13. How would you rate your overall well-being following the infusion compared with before the infusion? Substantially improved well-being Mildly improved well-being No change Worsening
